# Comparing different forms of structured follow-up support in the treatment of multiple health risks for chronic disease: a randomized controlled trial protocol

**DOI:** 10.1186/s13012-026-01527-x

**Published:** 2026-07-25

**Authors:** Emily R. Dunston, Amy G. Huebschmann, Ermyas Birru, Michaela Brtnikova, Bryan S. Ford, Rebekah Gomes, Kiana A. White, Andrea Nederveld, Demetria M. Bolden, Monica Perez Jolles, Elena T. Broaddus-Shea, Katy E. Trinkley, Debra P. Ritzwoller, Liza M. Creel, Jun Ying, Allison J. L’Hotta, Russell E. Glasgow

**Affiliations:** 1 Division of General Internal Medicine, Department of Medicine, University of Colorado Anschutz, Aurora, CO USA; 2 Adult and Child Center for Outcomes Research and Delivery Science, University of Colorado Anschutz, Aurora, CO USA; 3 Ludeman Family Center for Women’s Health Research, University of Colorado Anschutz, Aurora, CO USA; 4 Department of Pediatrics, University of Colorado Anschutz, Aurora, CO USA; 5 Department of Family Medicine, University of Colorado Anschutz, Aurora, CO USA; 6 Kaiser Institute for Health Research, Kaiser Permanente Colorado, Denver, CO USA; 7 Department of Physical Medicine and Rehabilitation, University of Colorado Anschutz, Aurora, CO USA; 8 Department of Medicine, University of Colorado Anschutz, Aurora, CO USA

**Keywords:** Cancer prevention, Whole person health, Follow-up support, Multi-behavior change, Navigation, Nudge, Implementation strategies, Pragmatic Robust Implementation and Sustainability Model (PRISM), Engagement, Costs

## Abstract

**Background:**

Primary care clinics need effective, low-cost tools to screen for and address multiple health risks — and technology can provide important efficiencies. My Own Health Report (MOHR) is a web-based intervention with 3 core functions: (1) risk flagging; (2) goal-setting for patient-prioritized risks; and (3) providing resources. A prior randomized trial in primary care settings showed that MOHR was feasible and effective. However, we identified *two key gaps*: a need for a flexible menu of delivery options to adopt and sustain MOHR and a pragmatic way to provide structured follow-up.

**Methods/design:**

We developed and pilot-tested two distinct *primary implementation strategies* to provide structured follow-up: the first primary strategy, termed “Reminder-Resource message (**R**^2^ **message)**,” involves automated motivational text message nudges to remind patients of their MOHR goal and point to resources. The second, **“R**^2^ **navigation**,” involves personal support - community health worker phone calls to deliver reminders and resources. Each primary strategy is paired with additional secondary strategies that together address the contextual determinants of successful implementation and sustainment identified using our Pragmatic Robust Implementation and Sustainability Model (PRISM). We engaged clinic staff, patients, and community partners to finalize the R^2^ message and R^2^ navigation strategies and secondary strategies. This individually randomized, type 2 hybrid effectiveness-implementation trial will enroll 1,000 adult patients with at least two cancer risks (including both insufficient physical activity and fruit/vegetable intake). We will evaluate the outcomes and cost-effectiveness of R^2^ message and R^2^ navigation alone or in combination in 12 primary care clinics including both (a) large metropolitan health system and (b) rural-serving clinics. Expected outcomes include: (1) improvement in behavioral risk factors; (2) implementation outcomes – including representative engagement with R^2^ message and R^2^ navigation, and cost; and (3) improvement in practice value outcomes, including patient experience ratings. Based on the trial findings, we will co-develop an implementation, adaptation and sustainment guide for the most cost-effective implementation strategy.

**Conclusions:**

We expect the implementation strategies compared will produce different levels of representative engagement, behavior change, cost, and practice value. These findings will inform pragmatic ways to improve outcomes important to patients, primary care, and society.

**Trial Registration# at ClinicalTrials.gov:**

NCT07569224 (Registration date: 04/29/2026).

Contributions to the literature

- Describes the co-development of an online health risk assessment and goal-setting tool integrated into the Electronic Health Record (EHR) to screen for and address multiple health risk behaviors.
- Experimentally tests the benefit of two primary implementation strategies that provide structured follow-up support: 1) Reminder and Resource (R^2^) message - automated motivational messages and resource recommendations versus 2) R^2^ navigation - community health worker phone calls and resource recommendations.
- Compares benefits of these two primary strategies – either alone or in combination – on the following: multiple risks for chronic disease (effectiveness); implementation outcomes – including cost; and value for primary care practices.

## Background

Many adults in the United States have at least one and often multiple risk factors for chronic illness (e.g., sedentary behavior, poor eating habits, high stress, social needs) [1–4]. Primary care is foundational in addressing these risks, given its focus on patient-centered care and longstanding partnerships to connect patients with resources to promote wellness [1, 4–7]. More recently, spurred on by recommendations from the National Academy of Sciences, Engineering and Medicine and others, primary care is shifting to a Whole Person Health Care approach [8–10]. Whole Person care proactively addresses multiple behavioral risks – prioritizing those that matter most to a given patient. Supporting patients to set goals to address multiple health behavior risks can feasibly reduce overall risk for cancer and other chronic diseases at the individual level. For primary care clinics, addressing multiple behavior risks may potentially improve blood pressure and laboratory values (e.g., hemoglobin A1c) that serve as quality metrics in primary care for hypertension and diabetes mellitus, respectively. Overall, this approach has great potential to generate value for individual patients and primary care practices.

**Fig. 1:**
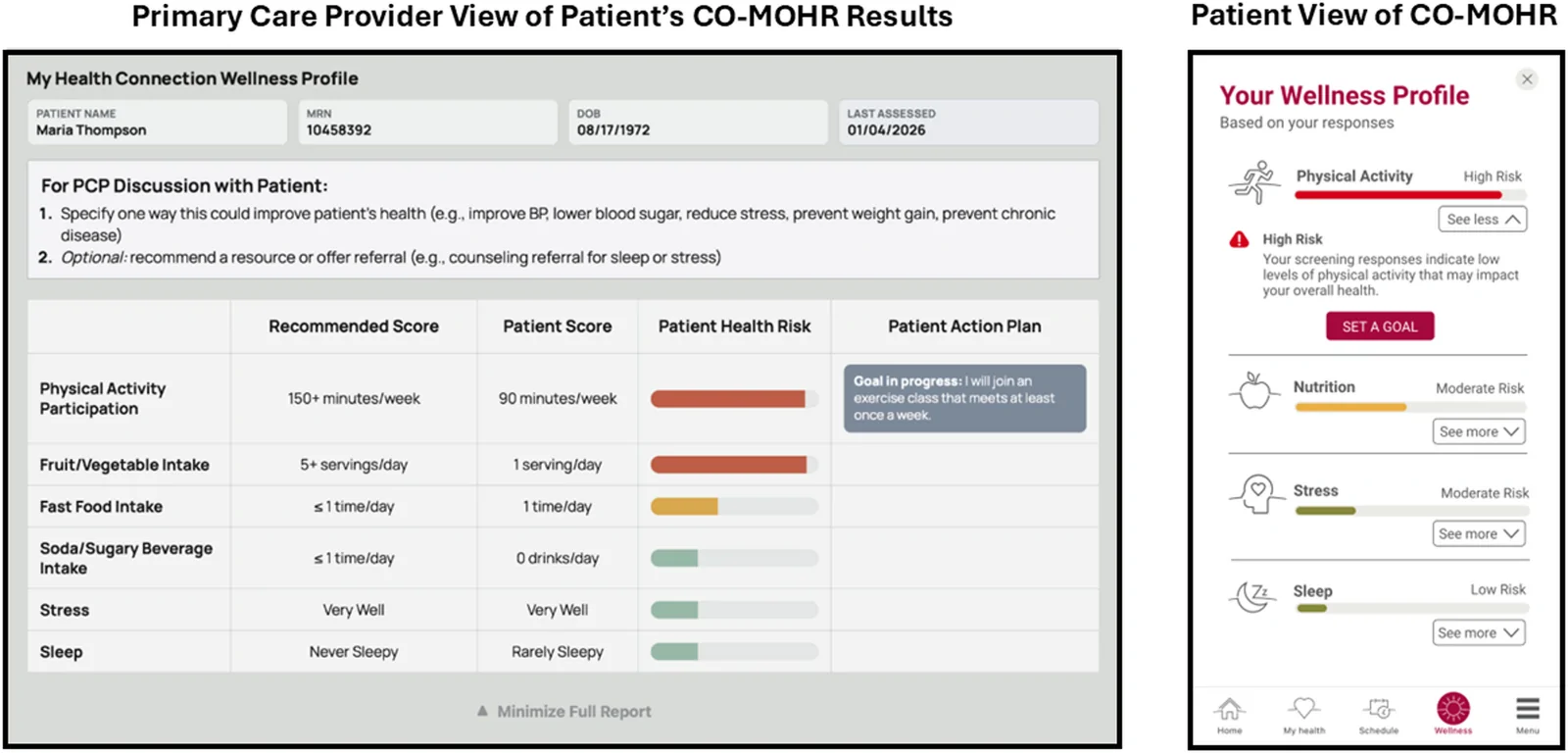
Colorado MOHR (CO-MOHR) feedback display for patients and primary care teams

*Clinics and patients value efficiency* - primary care providers desire assistance to address diet, physical activity, sleep and stress [1, 4, 11]. For efficiency, clinics should screen for and address **multiple risk behaviors simultaneously** ***- not just one issue at a time*** [1, 4]. To do this, our team has previously developed and tested the My Own Health Report (MOHR) intervention – and recently refined MOHR *to be delivered* through a freely available mobile application for the University of Colorado Health (UCHealth) System’s patient portal (hereafter termed *“app”*) [3, 4, 11–13]. The MOHR intervention has three core functions (i.e., theory/model-informed purposes) [14] drawn from the 5 A’s model [7, 15, 16] of behavior change: (1) *risk flagging*: identify and flag behavioral risks for cancer, (2) g*oal-setting*: patients review flagged risks and select from actionable goal options for a priority risk they are ready to change; and (3) *provide resources*: offer community or clinic resources for each behavioral risk in MOHR. After completing MOHR and setting a goal, a patient sees their health risk information in a simple, color-coded feedback display; for this Colorado MOHR (CO-MOHR) study, primary care teams can also see this display with prompts for discussion during a clinic visit (Fig. 1).

*Premise* – In our preliminary work, we found that patients in clinics randomized to the MOHR intervention vs. control significantly improved physical activity (*p* < 0.001) and diet risk behaviors (*p* < 0.001), as well as other risk factors more than usual care [3]. However, participating clinics voiced a need for a more flexible menu of delivery options to feasibly adopt and sustain MOHR to fit their varied clinic workflows (e.g., deliver ahead of visit through Electronic Health Record or during visit). Also, to achieve broad patient engagement and sustained behavior change, patients need to receive structured follow-up – this is particularly true among those with a higher burden of chronic disease and/or socio-economic need [7, 11, 12, 17–19]. Structured follow-up is part of the Expert Recommendations for Implementing Change (ERIC [20]) strategy of “*enhance uptake and adherence (among patients)*” - delivering this in an automated way also incorporates the ERIC strategy of “technical assistance.”

To fill these gaps, we completed a series of engagement activities. First, we worked with community, clinic and patient advisors to operationalize a “high tech” vs. “high touch” approach to balance pros and cons to our partners of personal tailored support (“high touch”) at higher cost with automated, low-cost support (“high tech”). Accordingly, we operationalized these structured follow-up strategies for this CO-MOHR intervention to be delivered either in an automated fashion (R^2^ message) or by a trained community health worker (R^2^ navigation) – see Table 1. Next, we worked with a subgroup of *primary care clinics and patient advisors* to co-develop a menu of delivery options for the CO-MOHR intervention in different practices and a menu of options for clinics to deliver our primary, randomized implementation strategies of R^2^ message and R^2^ navigation (Table 1). Specifically, from 2024 to 2026, we engaged with a subset of primary care clinics from a quaternary care academic health system (University of Colorado Health (UCHealth)) and a rural-serving practice-based research network as well as patient advisors to co-develop a menu of delivery options for the MOHR intervention and also a menu of options for our R^2^ message and R^2^ navigation strategies (Table 1).

The next section of this trial protocol manuscript provides details on the trial design and methods – including the primary strategies to be tested, as well as the analytic plan to evaluate the relative impact of each primary strategy on effectiveness, implementation outcomes, and value to clinics. We will also outline the procedures of a pilot trial to finalize the manual of procedures for the type 2 hybrid implementation-effectiveness trial [21–23]. These approaches will inform future research as well as implementation and sustainment decisions by primary care clinics.

## Methods/design

### Aims and hypotheses

**Fig. 2:**
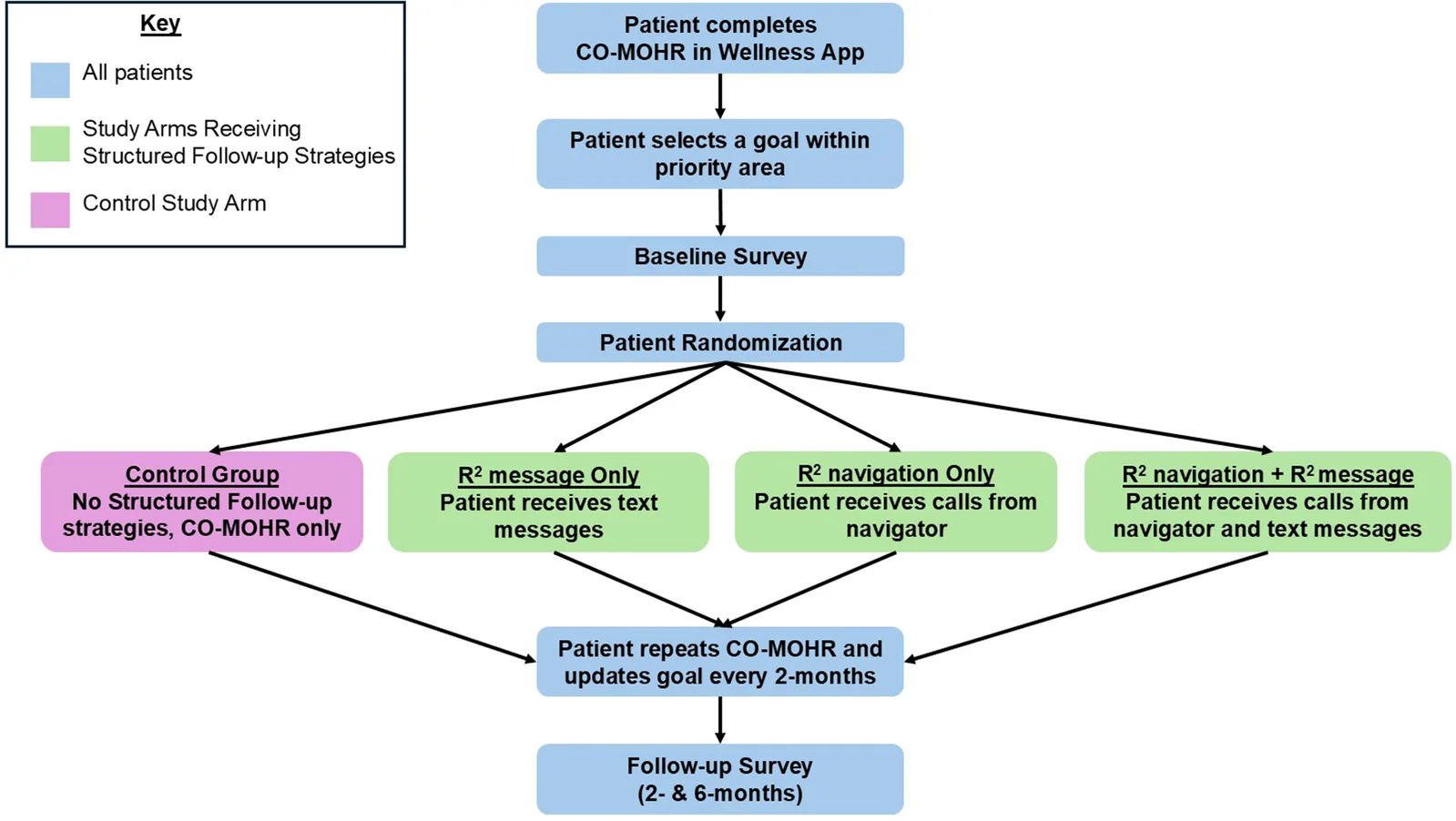
Schematic of patient flow through the study

Our primary aim is to compare the impact of distinct structured follow-up implementation strategies to support patients to achieve the goal they set in CO-MOHR: (1) R^2^ message, (2) R^2^ navigation, and (3) R^2^ message + R^2^ navigation as compared to a 4th group that receives the “usual” MOHR assessment and goal-setting without the additional structured follow-up (see Fig. 2). The benefits of these structured follow-up strategies will be assessed on multi-level outcomes of effectiveness (Table 3), implementation (Table 4), and practice value (Table 5).

The specific primary aim is: in a pragmatic comparative effectiveness-implementation trial, we will randomize *n* = 1,000 primary care patients with cancer risks of both low physical activity and low fruit/vegetable intake to test the impact on multi-level outcomes of three distinct options for structured follow-up for the CO-MOHR intervention as compared to a control condition of the CO-MOHR intervention with no structured follow-up. Secondary aims will identify which and how key multilevel contextual factors influence the representativeness of effectiveness, implementation, and sustainment outcomes using the Pragmatic Robust Implementation and Sustainability Model (PRISM).

These primary and secondary aims and the operationalization of the implementation strategies were finalized during the study pre-Implementation phase in co-creation processes with our partners, as briefly described above in the Background. Based on the findings of the primary aim and secondary aims in our trial, the final aim of this work will be to co-develop a CO-MOHR Implementation, Adaptation and Sustainment guide for the most cost-effective follow-up strategy. This dissemination guide will include information on implementation, adaptation, and sustainment so that other clinics can choose to implement MOHR in a way that fits the local clinical context.

**Fig. 3:**
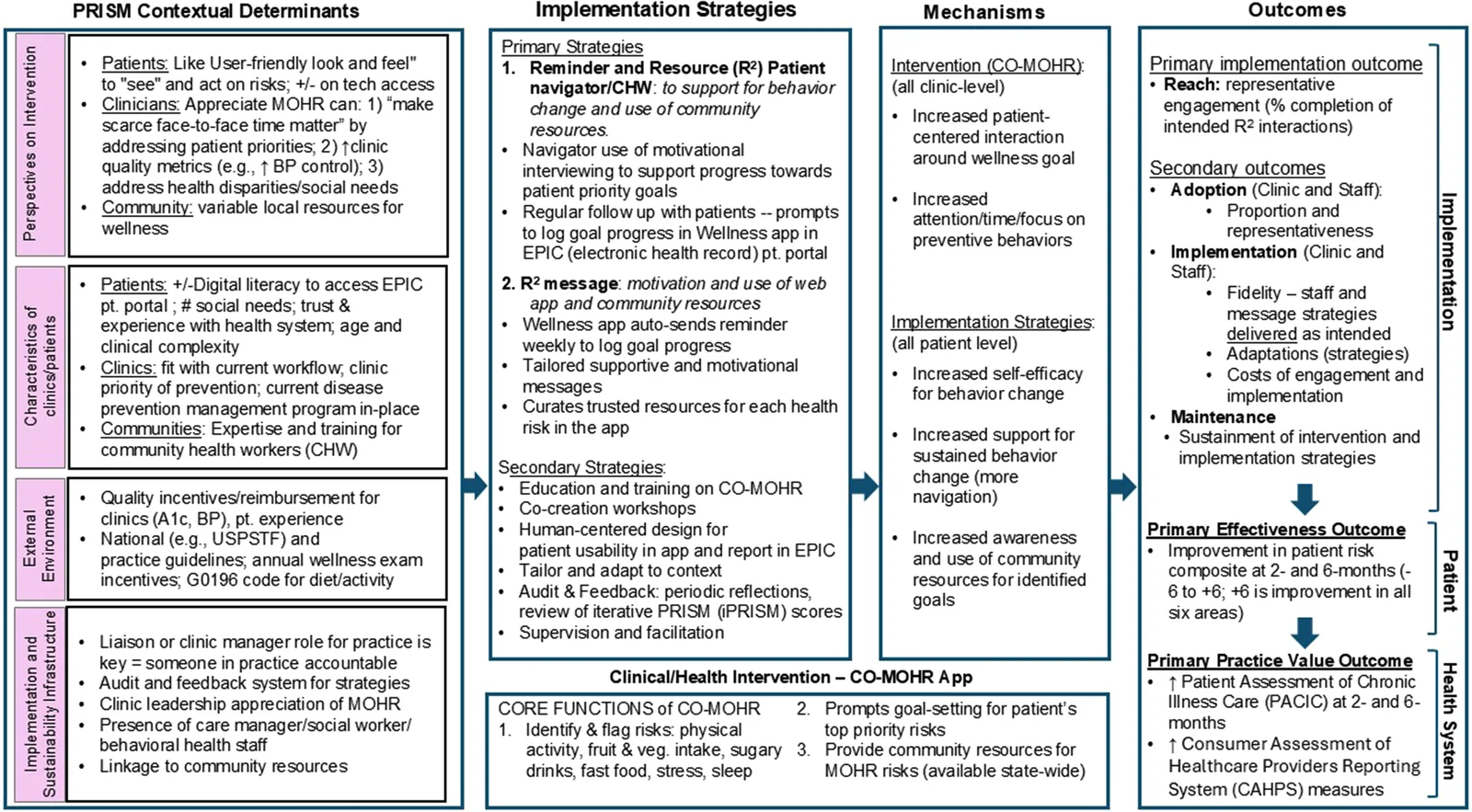
Implementation research logic model. Legend: A1c = glycated hemoglobin; BP = blood pressure; CO-MOHR = Colorado My Own Health Report Health Risk Assessment Intervention; MOHR = My Own Health Report; PRISM = Pragmatic, Robust Implementation and Sustainability Model; USPSTF = United States Preventive Services Task Force

The **expected outcomes** (overarching hypotheses) are that delivery of CO-MOHR with a structured follow-up strategy bundle will significantly reduce behavioral health risks for participants, as compared to the control condition, in part, due to higher self-efficacy and social-environmental support (see Fig. 3 for logic model). We also expect high levels of representative engagement – the rates of completion of the intended number of R^2^ message responses and R^2^ navigation visits, and improvements in the outcomes of value to the practice. In terms of the research question, we have equipoise for the differential impact of these implementation strategies (alone or in combination) on our measured outcomes, and expect the costs will be higher for the R^2^ navigation visits than for R^2^ message. The trial will identify the most cost-effective structured follow-up strategy, alone or in combination (i.e., Arm 1: R^2^ message, Arm 2: R^2^ navigation, and Arm 3: R^2^ message + R^2^ navigation), compared to the control group (Arm 4: usual care), to inform ways to assist a variety of primary care clinics to improve outcomes important to patients, primary care, and society.

### Study design

Prior to the type 2 hybrid comparative effectiveness implementation trial, we are conducting a **pre-trial pilot study** in Spring/Summer 2026 to beta-test and refine: 1) our recruitment strategies and survey procedures; 2) the interface of the co-developed CO-MOHR intervention with both the patient-facing “app” for the UCHealth system and the provider-facing electronic health record; 3) the system to deliver R^2^ text messages; and 4) manual of procedures for the R^2^ navigation calls by the community health worker (CHW). In this pre-trial pilot study, we plan to recruit patients who have a diagnosis of hypertension and/or diabetes (approximate n = 100) within UCHealth primary care clinics (n = 7).

We will launch the full type 2 hybrid comparative effectiveness implementation trial in Fall 2026 [21–23]. This is an individual-level randomized controlled trial; participant randomization will be stratified by clinic and patient ethnicity (Latine/Hispanic (L/H): Yes/No). Participants will complete the CO-MOHR intervention as part of usual care and then complete a baseline survey and provide postcard consent prior to randomization to a follow-up strategy condition. Within 5 business days of completing the baseline survey, **participants will be randomized to one of four study arms –** see Table 1 for the details on each primary strategy and Table 2 for the comparison arms tested: (1) R^2^ message, (2) R^2^ navigation, (3) R^2^ message + R^2^ navigation) and (4) usual care (control group).

**Table 1:** Preliminary operationalization of implementation strategies with menu of adaptations co-created with primary care partners

Primary Strategies	Implementation strategy elements, followed by preliminary menu of adaptations
Strategy 1: **R**^**2**^ **message ** MOHR app automatically *reminds* patients of goal and provides *resource* options	*Actor*: Automated text messaging system (Mosio^®^) – supervised by research team *Action*: Automatically sends message to patient’s cell phone with (1) motivational message (reminder) to address current goal and (2) encouragement to use community *resources* provided in CO-MOHR app to support goal attainment *Target*: patients randomized to R^2^ message *Timing/Dose*: daily for 4 weeks (20 messages); 3 days/week for 8 weeks (24 messages); 2 days/week for 4 weeks (8 messages); 1 day/week for 8 weeks (8 messages). *Preliminary menu of adaptations *[24, 25] Allowable adaptations for who enters preferred cell phone number into MOHR: by patient or staff (with patient permission) *Representativeness considerations*: R^2^ message provided in both English and Spanish
Strategy 2: **R**^**2**^ **navigation** Calls by a navigator to revisit goal, update action plan and provide resource options	*Actor*: Navigator - certified community health worker, trained in Motivational Interviewing *Action*: Navigator contacts patient with (1) reminder of goal and (2) encourages use of community resources from CO-MOHR app and (3) serves as an action plan resource – uses Motivational Interviewing to discuss action plan options for patient’s goal *Target*: patients randomized to R^2^ navigation *Timing/Dose*; 1 call/week for 4 weeks (4 calls); 1 call every other week for 12 weeks (6 calls); 1 call every 4 weeks for 8 weeks (2 calls) = 12 calls in total *Preliminary menu of adaptations *[24, 25] Allowable adaptations in mode of contact for navigator: phone call, video call *Representativeness considerations*: Bilingual (English/Spanish) Navigators

*ERIC [20] strategies include: Enhancing uptake and adherence with patients; Technical assistance – for R^2^ message this assistance is from the automated message; for R^2^ navigation the assistance is from the navigator

### Implementation science framework

We are using PRISM to guide the development and evaluation of this hybrid type 2 implementation effectiveness trial [21–23]. PRISM was selected because it is pragmatic with a relatively small number of key contextual factors to assess and yet considers multi-level perspectives. Importantly, PRISM includes both contextual determinants of implementation and the Reach, Effectiveness, Adoption, Implementation and Maintenance (RE-AIM) implementation outcomes [26–28]. The four contextual domains in PRISM are: (1) the perspectives of the patients and implementers on the intervention (and implementation strategies), (2) the characteristics of the clinics and patients, (3) the external environment and (4) the implementation and sustainability infrastructure [26–28]. External environment includes policies, incentives, and regulations that impact the implementation of CO-MOHR. The implementation and sustainability infrastructure in this study include resources and processes such as audit and feedback and staff with dedicated roles to support implementation and maintenance.

### Implementation research logic model

Our implementation research logic model [29] (Fig. 3) outlines our key PRISM findings concerning contextual factors from our preparatory engagement work on the left-hand side, then the implementation strategies that we will test, the core functions of the CO-MOHR intervention, expected mechanisms through which the intervention and strategies will impact outcomes, and our multi-level RE-AIM outcomes [26, 28]. 

As described in Fig. 3, the PRISM contextual factors identified in our engagement work provide both barriers and facilitators to successful delivery of MOHR. The two primary implementation strategies tested were described in Table 1. As with any program, there are also secondary implementation strategies such as education and training that are common to all study arms. The primary mechanisms expected a priori are separated by the CO-MOHR intervention and the implementation strategies, and the RE-AIM outcomes outlined in the logic model are described in detail in Tables 3, 4 and 5.

### Colorado MOHR health risk assessment intervention (CO-MOHR)

The CO-MOHR intervention is a Whole Person Health-focused [8–10] assessment and goal-setting tool that has been tested and refined through delivery in primary care clinics [11, 12, 30]. MOHR was originally designed as an external website; in this study, the CO-MOHR intervention will be embedded into the patient-facing app. The three core functions of CO-MOHR draw on the rich development of MOHR, as described in the Background, these three core functions are consistent across study arms: (1) risk flagging, (2) goal-setting, and (3) provide resources to meet CO-MOHR goals. Risk flagging involves the app identifying behavioral risk factors for developing chronic diseases such as cancer. The health behavior risks included in CO-MOHR were selected based on input from patient advisory boards, primary care clinic teams, and the patient experience team involved with the app build. The six health behavior risks CO-MOHR includes are: (1) low physical activity, (2) low fruit and vegetable intake, (3) high sugar sweetened beverages consumption, (4) high unhealthy fast-food consumption, (5) high stress, and (6) low sleep. Goal-setting involves the patients reviewing flagged risk areas in the app and selecting one of three actionable goal options for the risk they are ready to change. Providing resources involves display in the app of free or low-cost resources to support goal attainment.

### Primary implementation strategies (Comparators)

The primary implementation strategies in this study were co-developed in the trial planning phase with participating primary care clinics and patient advisors. The key functions of the primary implementation strategies, R^2^ message and R^2^ navigation, are summarized in Table 1. Briefly, the R^2^ message only group (Arm A) will receive motivational text messages specific to their goal topic, based on Self-Determination theory [31] and their level of progress reported. The R^2^ navigation only group (Arm B) will receive repeated coaching sessions (6 in the first 2 months and another 6 in the next 4 months) over the phone from a wellness coach trained in motivational interviewing techniques and certified to meet the standards to serve as a community health worker in Colorado [32, 33]. Each call will follow a structured outline — described in a manual for each community health worker’s reference. The R^2^ message + R^2^ navigator group (Arm C) will receive both follow-up strategies simultaneously.

### Study setting & partnerships

Our primary care clinic partners include seven University of Colorado Health (UCHealth) clinics in the Denver, CO, USA metropolitan area and five clinics in a practice-based research network in Western Colorado that serves small metropolitan and rural populations. The implementation blueprint for CO-MOHR was adapted through co-design using the Functions and Forms approach (i.e., activities tailored and/or adapted to local contexts) [14] to the resources and needs of the different clinic types. See Table 1 for a preliminary menu of allowable adaptations for each primary implementation strategy – requests for allowable adaptations to implementation strategies will be reviewed by the MPIs and added to the manual of procedures if allowable without permission (green light), allowable with permission (yellow light) or not allowable (red light) [34]. Additionally, we are partnering with a community health worker organization (Vuela for Health) to deliver the R^2^ navigation visits – their community health worker staff are Latine promotoras, experienced in health promotion, and trained in Motivational Interviewing as certified community health workers in Colorado [32, 33]. 

### Recruitment

#### Primary care clinic recruitment

We invited a total of 17 primary care clinics (UCHealth Urban/Suburban: *n* = 10; Western Colorado Rural-serving: *n* = 7) to participate in this study. Inclusion criteria for primary care clinics included: (1) adult primary care clinics; (2) clinics that engaged with our team as part of the UCHealth system or as part of a practice-based research network [6]. Participating clinics expressed a desire to prioritize inviting patients with type 2 diabetes and/or hypertension to complete MOHR, aligned with the clinic’s need to improve those quality metrics. The *n* = 5 clinics that declined to participate cited competing demands.

#### Patient recruitment

Eligibility criteria for patients included: (1) age of ≥ 18 years, (2) patient at a participating primary care clinic, (3) completion of CO-MOHR assessment as part of standard of care, and (4) CO-MOHR identified health behavior risks for both physical activity and diet. Exclusion criteria included: (1) language preference other than Spanish or English, (2) no or incomplete CO-MOHR assessment, and (3) diagnosis of dementia/taking medications for dementia. Patients who meet eligibility criteria will be identified using an EHR-based recruitment tool and sent the baseline survey electronically with a postcard consent describing the potential follow-up messages or calls they may receive. This consent process was approved by the Colorado Multiple Institutional Review Board (COMIRB # 25–0224 (on June 18, 2025)) and by the UCHealth system, with the condition to give clear opportunities to “opt out” from messaging at any time. The COMIRB review was in accordance with the ethical standards of the Declaration of Helsinki. Following baseline survey completion, patients are assigned to one of the four study arms described below (Table 2).

**Table 2:** Comparisons by study arm

Outcome level (Type)	Comparison tested
Effectiveness - change in risk factors	Pair-wise comparisons: A vs. B; A vs. C; B vs. C
Implementation- representative engagement	Pair-wise comparisons: A vs. B; A vs. C; B vs. C
Practice Value - patient experience	Compare all: A vs. B vs. C vs. D
Compare CO-MOHR intervention with one or more structured follow-up strategy (A, B, and C) vs. Control (Arm D) from baseline to 2-months (Primary outcome time-point) and 6-months	

Note: The four study arms are defined as

A: R^2^ message (motivational text messages addressing the patient’s priority goal)

B: R^2^ navigation (phone calls addressing the patient’s priority goal

C: Both R^2^ message and R^2^ navigation

D: MOHR intervention alone (Control - no structured follow-up strategy = no motivational text messages or calls)

### Outcomes/data collection

We will assess outcomes quantitatively and qualitatively using a mixed-methods approach. Following a description of the data collection approaches, we then present the primary and secondary outcomes for: effectiveness (Table 3), implementation outcomes (Table 4), and practice value outcomes (Table 5).

### Quantitative and qualitative data collection

Data collection methods and procedures include tracking of outcomes and tasks, surveys, and interviews.

- Task tracking will be ongoing to inform assessments of costs; research study team members and navigators will track tasks and time spent across all study arms.
  - Tracked outcomes include number of contacts and time spent delivering the CO-MOHR intervention, delivering messages and navigation calls, respectively, as well as time spent by study team and clinic partners on engagement and implementation-related activities.
- Follow-up surveys will be sent as follows.
  - All participants (primary care patients): Automated survey links sent from a HIPAA data-compliant online database at baseline, 2-month and 6-months – each link will include up to 2 follow-up contacts for completion. Survey items are tailored to the target population and outcome assessed and are defined in more detail for each RE-AIM outcome in Tables 3 and 4, and 5.
  - Navigators: Completion of the iterative PRISM (iPRISM) Webtool at least twice yearly. The iPRISM Webtool is a web-based survey to assess PRISM factors and is completed by all team members to assess alignment on implementation strategies with their context and progress on RE-AIM outcomes [35].
- Periodic reflections [36] will be conducted at least 3 times yearly with navigators and 1–2 times yearly with a purposive sample of primary care clinic team members from each participating clinic. These conversations will explore PRISM contextual domains, such as perceptions and experiences with the program.
- Check-in/supervision meetings will be conducted with navigators every 1–3 weeks to assess perceptions, experiences, adaptations, and challenges with patient navigation, and project workflow.
- Interviews will be conducted with a purposive sample of patients immediately after completion of the 2-month follow-up survey and again after the 6-month follow-up survey.
  - Interviews will explore topics of acceptability, appropriateness, and perceptions of the program, experiences with program reach, intervention and implementation strategy delivery quality, adaptations, effectiveness, and reasons for continuing the program or not.

### Primary effectiveness outcome: changes in the number of MOHR risk factors

CO-MOHR is organized into four domains (Nutrition, Physical Activity, Stress, Sleep) that include a total of six distinct health behavior assessments: (1) fruit and vegetable intake, (2) sugar-sweetened beverage consumption, (3) fast food consumption, (4) physical activity, (5) stress, and (6) sleep. Risks are self-reported using the CO-MOHR survey at baseline, 2- and 6-months using validated cut points for low, moderate, and high risk on items as reported previously [3, 30]. The primary effectiveness outcome assesses: change in total number of health behavior risks from baseline, calculated as number of risk factors showing any level of improvement minus the number of risk factors showing any negative change (range − 6, + 6), with 2 months as the primary timepoint.

### Secondary effectiveness outcomes: physical activity and fruit & vegetable consumption

Physical activity will be assessed on the CO-MOHR survey using the exercise vital sign [37, 38] — a self-report of the number of days over the past week and average minutes per day spent in moderate-to-vigorous intensity physical activity. Fruit and vegetable consumption will be assessed by a self-reported number of servings of fruits/vegetables consumed each day over the past seven days. Change in physical activity and fruit and vegetable consumption from baseline will be calculated at 2- and 6-months follow-up (see Table 3 for details). In addition to these primary and secondary outcomes, we will assess exploratory outcomes (see Table 3).

**Table 3:** Multi-level effectiveness outcomes

Measure Description	Quantitative Assessment	Timing	Qualitative Assessment
**Primary Effectiveness Outcome**			
Change in number of CO-MOHR risk factors	Number of risk factors changed measured on scale from − 6 to + 6.* CO-MOHR questions are organized into 6 risk areas: (1) intake of fruits and vegetables; (2) high sugar beverages; (3) fast food; 4) physical activity; 5) stress; and 6) sleep.	Change (∆) from baseline to 2-months (primary) and 6- months (secondary)	Patient interviews – understand PRISM contextual influences on overall number of risks, and changes in specific risk factors
**Secondary Effectiveness Outcomes**			
Physical Activity	Physical activity vital sign – physical activity in minutes/week	∆ from baseline survey to 2- and 6-months	Patient interviews, understand PRISM context for physical activity
Fruit and Vegetable Intake	Number of fruit/vegetable servings per day	∆ from baseline survey to 2- and 6-months	Staff periodic reflections – PRISM factors influencing implementation
**Exploratory Outcomes**			
Tobacco Use	Tobacco use in the last 30 days (yes/no; frequency of use) [39] and Reported quit attempt since last assessment (yes/no)	∆ from baseline survey to 2- and 6-months	Patient interviews, understand PRISM context for tobacco use
Alcohol Use	Frequency of alcohol use and typical number of standard drinks consumed	∆ from baseline survey to 2- and 6-months	Patient interviews, understand PRISM context for alcohol use
Unmet Social Needs	Number of unmet social needs – food insecurity, medication affordability, lack of transportation, financial problems related to medical care	∆ from baseline survey to 2- and 6-months	Patient interviews, understand PRISM context for social needs

*Each risk factor is assessed on a three-level ordinal scale (high, moderate, low). Improvement in a given risk area is defined as a decrease in risk level over time (high to moderate, high to low, or moderate to low). The primary effectiveness outcome will be a numerical score from − 6 to 6 (any improvement or decrement in level of risk in each factor will count as + 1 or − 1, respectively) number of risk areas (out of six) in which change is observed. CO-MOHR = Colorado My Own Health Report; PRISM = Pragmatic, Robust Implementation and Sustainability Model

### Primary implementation outcome: representative reach

Reach of CO-MOHR, defined as representative engagement for this trial, will be operationalized as the percent of intended implementation strategy interactions completed (text message responses and/or navigator calls completed) during the first 2 months of intervention (primary) and at 6 months (secondary). To assess whether reach is representative, we will examine engagement across: (1) ethnicity, (2) level of baseline social needs, and (3) digital literacy. An additional exploratory measure of representative engagement is the number of “check-ins” in the CO-MOHR app for each goal set.

### Secondary implementation outcomes: implementation and sustainment

Following RE-AIM definitions [40–42] Implementation outcomes will include fidelity, adaptation, costs and representative drop out. Costing and economic analyses are described separately below. Fidelity will include automated operational data from CO-MOHR of goal-setting, delivery of and response to motivational messages, activities completed as well as adaptations made for each session on navigator checklists, and recording/review of 5% of navigator calls. Periodic reflections [36] with navigators and clinic staff will also inform fidelity and adaptations. Adaptations will be documented using FRAME-IS supplemented with RE-AIM issues and include estimated impact [24, 43, 44] via operational data and periodic reflections. Adaptations will be tracked continuously and summarized during navigator check-in meetings/periodic reflections, and reviewed quarterly. For representativeness, dropout rates will be assessed across the four study arms, and across clinics, navigators, and demographic characteristics.

Sustainment will be assessed at both the setting and patient levels. At the setting level, sustainment will be defined as the percent of clinical sites that: (1) continue to offer CO-MOHR after trial completion with either R^2^ message alone, R^2^ navigation alone, or R^2^ message + R^2^ navigation, or (2) continue CO-MOHR after trial completion with modifications. In addition, clinic/health system sustainment will be assessed using the Moullin Sustainment Scale [45]. Patient level sustainment (individual-level maintenance in RE-AIM) will be assessed by health outcomes at 6- months.

**Table 4:** Multi-level implementation outcomes

Measure Description	Quantitative Assessment	Timing	Qualitative Assessment
**Primary Implementation Outcome**			
Reach – Representative Engagement	Engagement during the follow-up period involves interactions over time of the participant with either or both a navigator or text messages. Engagement will be assessed as percent of intended contacts completed* Assessment of if engagement is representative will be assessed by engagement across conditions (e.g., an interaction effect) based on (1) number of baseline social needs, (2) Latine vs. non Latine ethnicity or (3) digital literacy.	2-months (primary time point) and 6-month period (secondary time point) following randomization	Patient interviews, reasons for accepting or discontinuing R^2^ message and/or R^2^ navigation
**Secondary Implementation Outcomes**			
Fidelity	Fidelity to primary implementation strategy blueprints based on MOHR online platform data on topics addressed (e.g. check on progress, goal setting, delivery of motivational messages) and Navigator checklists on completion of these actions	Over 2 months from randomization (primary) and 6 months (secondary	Staff periodic reflections – PRISM (Staff perspectives/characteristics domains - factors influencing implementation)
Representative Drop Out	Differential drop out – drop out across conditions and patient characteristics.	At 2 months (primary) and 6 months (secondary	Staff periodic reflections – PRISM factors influencing implementation
Adaptations	Adaptations assessed using FRAME-IS via program tracking records and periodic interviews of participating clinic staff	Over 2 months (primary) and 6 months (secondary)	Staff periodic reflections – PRISM factors influencing implementation
Sustainment (Maintenance in RE-AIM terminology)	*Setting level − 1*) Percent of sites that continue to offer MOHR with messages and/or navigation, modify delivery, or stop 2) Moullin sustainment scale	At 6 months (and possibly longer time periods if additional funding is acquired)	Staff interviews – PRISM factors influencing sustainment

Note: MOHR = My Own Health Report; RE-AIM = Reach, Effectiveness, Adoption, Implementation, and Maintenance; PRISM = Pragmatic, Robust Implementation and Sustainability Model

### Primary practice value impact outcome: patient experience

Change in the mean score for the “goal-setting/tailoring” and “follow-up/coordination” subscales of the Patient Assessment of Chronic Illness Care (PACIC) [46] adapted to preventive behaviors will be used to evaluate patient experience. Change in PACIC subscale scores (quality of care) from baseline will be assessed via 2-month (primary), and 6-month follow-up surveys.

### Secondary practice value impact outcomes: cost and patient experience

A single item from the Consumer Assessment of Healthcare Providers Reporting System (CAHPS) survey [47] that assesses patient experience will be used to supplement the primary patient experience outcome. This is widely used by clinics nationally as a performance measure and is thus of value to participating clinics. Change in patient experience, as measured by CAHPS, from baseline will be calculated at 2- and 6-months follow-up. Cost and cost-effectiveness will be determined by time-based micro-costing approaches (see Economic Analysis Plan below for additional details).

**Table 5:** Multi-level practice value outcomes

Measure Description	Quantitative Assessment	Timing	Qualitative Assessment
**Primary practice value outcome**			
Patient Experience	Change in mean score on Patient Assessment of Quality-of-Care subscales of goal setting/tailoring and follow-up/coordination (PACIC)	Change (∆) from baseline (survey) to 2-months (primary) and 6-months	Patient interviews – PRISM (patient perspectives domain)
**Secondary Outcomes**			
Consumer Assessment of Healthcare Providers Reporting System (CAHPS)	Standardized CAHP 4-item Likert scale question on patient experience used to evaluate primary care quality	∆ from baseline (survey) to 2-, months (primary) and 6 months	Patient interviews – PRISM (patient perspectives domain)
Costs of (1) engagement and (2) time and resources to deliver implementation strategies– and any reimbursement	Time-based activity micro-costing of staff time required overall and per patient, including time required for engagement (e.g., co-creation, securing approvals) as well as implementation strategies. We will also assess time spent by navigators and track any insurance reimbursement for navigation	Assessed by condition at 2-months (primary), and 6-months	Staff periodic reflections – PRISM contextual factors (staff perspectives and characteristics domains, factors influencing implementation)

Note: PRISM = Pragmatic, Robust Implementation and Sustainability Model

### Statistical power and analysis plan

All variables, including the outcome measures and baseline characteristics, will be summarized using descriptive statistics for each group and each time point. Distributions of outcomes will be assessed for normality. If the assumption of normality is not met, appropriate alternative distribution assumptions will be considered. We will monitor the missing values closely and use appropriate analytic approaches based on the types of missingness (e.g., likelihood-based method, multiple imputation, or pattern mixture models). For any assumptions of missing condition or mechanism, sensitivity analyses will be performed to ensure the findings are robust.

### Power calculations

All sample size and power calculations were conducted using two-sided tests with an overall α = 0.05. Multiple comparisons between study arms at a given follow-up time point, and across time points within an arm, will be adjusted using the Bonferroni method. Table 6 below summarizes the statistical power to detect differences based on different assumptions about sample size, attrition, and the type of outcome.

We plan to recruit and randomize 1,000 participants (*N* = 250 per arm) and anticipate retaining approximately 800 participants (*N* = 200 per arm), assuming an attrition rate of up to 20%. The primary effectiveness outcome is the change of number of risk factors identified by MOHR, defined as a numerical variable. As can be seen below, with 800 evaluable participants, the study will have 80% power to detect an effect size of 0.33 (defined as difference in changes divided by the standard deviation of the difference) when comparing changes among study arms at a given follow-up time point. The same power calculations apply to the primary implementation outcome of representative engagement and the primary practice value outcome of patient experience.

Table 6 also summarizes power for other sample sizes and different types of variables since one of the outcomes above may fail to meet normality assumptions, and some secondary and exploratory outcomes are binary variables.

**Table 6:** Summary of power analysis

Type of Comparison	Total N at Enrollment	Total N after Attrition	N/Group	Effect Size for Continuous Variable*		λ1/ λ0 for Count Variables**		Odds Ratio for Binary Variables***	
				Power 80%	Power 90%	Power 80%	Power 90%	Power 80%	Power 90%
Comparison between control and intervention groups	800	640	160	0.37	0.41	85.80%	83.90%	0.48	0.44
	1000	800	200	0.33	0.37	87.20%	86.50%	0.52	0.48
	1200	960	240	0.30	0.34	88.30%	86.70%	0.55	0.51
	1400	1120	280	0.28	0.31	89.10%	87.70%	0.57	0.53
	1600	1280	320	0.26	0.29	89.80%	88.50%	0.60	0.56
Comparison between baseline and follow-up timepoint	800	640	160	0.27	0.30	90.60%	89.25%	0.62	0.57
	1000	800	200	0.24	0.27	91.55%	90.35%	0.66	0.62
	1200	960	240	0.22	0.25	92.28%	91.17%	0.67	0.63
	1400	1120	280	0.20	0.23	92.82%	91.78%	0.70	0.66
	1600	1280	320	0.19	0.21	93.28%	92.32%	0.72	0.68

† All power analyses are based upon proposed statistical models using two-sided tests. Multiple comparisons are adjusted for using Bonferroni’s method

* Effect size is defined as the difference of means/standard deviation of difference

** λ1 denotes the mean count in an intervention group or in a follow up time point

*** Odds ratio denotes the ratio of odds between an intervention group against the control group at a time point, or the ratio of odds between a follow up time point against baseline for a group

### Quantitative analysis

For outcomes with repeated measurements, associations with the fixed effects of interest (implementation strategy, time, and their interaction) will be evaluated using generalized linear mixed-effects models (GLMMs). Depending on the outcome type, models will assume a normal distribution with an identity link (numerical outcome), a Poisson distribution with a log link (count outcomes), or a binomial distribution with a logit link (binary outcomes). Fixed effects will include implementation strategy, time, and their interaction. A random intercept for participants will be included to account for within-person correlation due to repeated measurements.

Post hoc comparisons will be conducted between study arms at a given follow-up time point and across time points within an arm, with adjustment for multiple comparisons using the Bonferroni method. These comparisons will be expressed as mean differences (numerical outcomes).

The GLMMs will allow adjustment for potential confounders and covariates. Baseline characteristics (e.g., sociodemographic factors, health conditions and behaviors, and clinic characteristics) will first be examined for associations with outcomes using generalized linear models (GLMs), and for associations with implementation strategy using appropriate methods (e.g., ANOVA for continuous variables and chi-square tests for categorical variables). Variables showing statistically significant associations in either analysis will be considered candidate confounders and included as covariates in the GLMMs. Results from post-hoc estimates and comparisons will be reported from both unadjusted and adjusted GLMMs.

#### Moderator analysis

Analyses will be performed to inspect if the findings from the primary analysis are sensitive to the subpopulations stratified by language, clinic, and presence/absence of diabetes. For each candidate moderator, its effect will be assessed by adding the moderator and its interactions with the key fixed effect of interest as additional fixed effects in the GLMMs and GLMs.

### Qualitative analysis

After each round of interviews and periodic reflections, we will conduct a rapid qualitative analysis [48, 49]. We will complete a summary template for each data collection episode covering key domains of interest, and compile these into a matrix to facilitate identification of themes and trends within and across domains. Finally, we will use a mixed methods convergent design [50] to integrate quantitative and qualitative findings. Quantitative and qualitative data will be collected and analyzed separately. These two sets of data will then be analyzed and interpreted together using joint display analysis [51, 52]. We will examine the data for concordance/discordance, especially related to differential patterns of representative reach and/or effectiveness.

### Economic analysis

An economic evaluation of the MOHR implementation and intervention will assess the variation in cost and cost-effectiveness of the four study arms (Table 2). Cost-effectiveness will be measured in terms of the incremental resources and costs relative to the usual care condition, for each of the other 3 study arms, per the incremental change in the primary outcome. This metric is often referred to as an *incremental cost-effectiveness ratio* (ICER). The primary outcomes used to estimate the ICERs will be the average incremental change in (a) the Primary Effectiveness outcome (change in number of MOHR risk factors); and (b) the Primary Practice Value outcome – the sum of two key Patient Assessments of Chronic Illness Care (PACIC) [46] sub-scales for each of the 4 MOHR arms. No patient identifiable information will be included.

Given that the resources and costs needed for the implementation strategies are largely driven by differences in variable resources including navigator time and other staff time (including training), materials and supplies, and other fixed resources (e.g., app updates, maintenance), we will use methods and instruments consistent with our previous studies, along with novel strategies recently developed in our previous Colorado Implementation Science Center for Cancer Control and Prevention grant and Anschutz Acceleration Award [53, 54]. We will also evaluate the costs of multi-source partner engagement [54]. These methods will guide evaluation of the incremental differences in the resources and the costs required for all study arms. Based on our goals to further adoption and sustainment, we will consider both the practice and the payer perspectives. Resources will be captured by the following methods:

1. Brief structured interviews with clinic implementation staff using process maps created for each clinic that identify who does what, when, and how long that activity takes, using methods from our prior health behavior change projects [55, 56]. These interviews will cover activities involved in initial identification of patients, completion of MOHR, discussion with patients during primary care visits about priority goals, and delivery of the R^2^ message texts and/or R^2^ navigator calls.
2. Time capture instruments and meeting records will document time spent engaging with clinic leaders, approval committees, and patient advisory boards. These tools will document time of the research team and these partner groups and any other resources (e.g. participant incentives) that facilitate engagement.
3. Research staff records of time to train and supervise navigators.
4. Logs of navigator time spent contacting patients in R^2^ navigation arms.
5. Time spent developing and delivering the R^2^ message arm.
6. Measures of patient MOHR app usage and text message review to derive proxies of patient time.

Resource use associated with the intervention and implementation strategies will be valued at competitive market rates using standard job classifications required for noted tasks, along with “make versus buy” scenarios for technology resources used for the website, messaging etc. [57]. We will capture all planning, engagement, recruitment, intervention, and implementation attributable expenses using procedures that will allow us to differentiate research and development costs, CO-MOHR intervention costs, and implementation strategy costs - data that will be useful for informing decisions related to sustainment and future dissemination. We will also differentiate recruitment costs and participant incentives from the intervention and implementation strategy costs. We will capture both fixed costs (e.g., development of web application, training tools), and patient volume-based variable costs (e.g., navigation). These time and resource cost estimates will be aggregated with and across each implementation strategy to calculate the incremental improvement in the relevant outcome. For example: we will report on the costs for the R^2^ navigation strategy for the incremental change in number of risk factors and PACIC [46] scores, as compared to other conditions. We will report the costs with sensitivity analyses for variability across sites and will track reasons for variability [58]. Finally, using procedures we have pioneered, we will estimate replication costs to deliver the resulting primary implementation strategy in different settings with different EHRs and Navigator roles [59–61]. This analysis will provide key information regarding the most cost-effective primary implementation strategy across the study arms to develop a MOHR Implementation, Adaptation and Sustainment Guide.

### Implementation guide for dissemination

We will co-develop, with implementation partners, a MOHR *Implementation*,* Adaptation and Sustainment Guide* for the most cost-effective primary implementation strategy from this trial with attention to representative reach and clinic sustainment. This guide will include links to resources needed to implement the program, and potential sources of revenue to support implementation (e.g., G0136 billing for diet and physical activity assessment/counseling). By disseminating this guide through primary care societies, we will equip primary care clinics to improve and sustain outcomes important to patients, primary care, and society.

## Discussion

This research is being conducted as part of an electronic health record-embedded trial [62] at the University of Colorado Anschutz. Accordingly, there are several feasibility constraints that needed to be considered. This approach to embedding the intervention within a quaternary health system’s patient portal has strengths of allowing us to obtain real-world evidence regarding the structured follow-up implementation strategies tested. To obtain real-world data, we also needed to follow feasibility constraints, such as constraining the number of Wellness Profile domains overall to six risk factors across the four high-priority wellness domains for the health system’s Digital Patient Experience team: nutrition, physical activity, sleep and stress – these are also featured in other national health risk assessments such as Life’s Essential Eight by the American Heart Association [63]. 

In terms of expected outcomes, we expect to reduce multiple health risks for cancer and other chronic disease and to observe high levels of representative engagement across the R^2^ message strategy (Arm A), R^2^ navigator strategy (Arm B), both strategies (Arm C) as compared to the control group (Arm D). Many of the potential implications of our findings relate to the primary implementation strategy that is identified as most cost-effective. If we find that the low-cost R^2^ message is the most cost-effective structured follow-up strategy, our team will seek additional funding to test the dissemination of building MOHR with follow-up support text messaging into other health systems. If the R^2^ navigator is the most cost-effective structured follow-up strategy, it would justify expansion of payments for community health workers that are presently covered by payment models such as Principal Illness Navigation by the Centers for Medicare and Medicare Services. Overall, we expect the lessons learned from this trial will position primary care clinics to innovatively support patient-centered approaches to address “what matters most” to a given individual to improve their risk for cancer and other chronic diseases.

## Data Availability

No datasets were generated or analysed during the current study.
